# Nursing Practice Environment, Work‐Family Conflict, and Turnover Intention Among Female Nurses With Children Under 18 years of Age in Japan: A Nationwide Cross‐Sectional Study

**DOI:** 10.1155/jonm/5439285

**Published:** 2026-07-31

**Authors:** Liyuan Zheng, Hiromi Kawasaki, Wenjie Guo, Miyuki Toda, Yoko Shimpuku

**Affiliations:** ^1^ Graduate School of Biomedical and Health Sciences, Hiroshima University, 1-2-3 Kasumi, Minami-ku, Hiroshima City, Hiroshima, Japan, hiroshima-u.ac.jp

**Keywords:** female nurses, nursing practice environment, turnover intention, work–family conflict

## Abstract

**Objective:**

To investigate the levels of the nursing practice environment, work–family conflict, and turnover intention and explore the mediating role of work–family conflict between the nursing practice environment and turnover intention among female nurses with children under 18 years.

**Methods:**

An online survey recruited 305 registered female nurses from 45 prefectures across Japan between July and September 2025. Variables were measured using demographic data, scales of maternal feeling of childcare burden, the practice environment of the nursing work index, work–family conflict, and turnover intention. Data analyses were performed using one‐way analysis of variance, *t*‐tests, Pearson correlation analysis, multivariate linear regression analysis, and the Process 4.0 macro plug‐in method (SPSS v. 26.0).

**Results:**

Turnover intention among female nurses differed significantly according to their cohabitation status, youngest child’s age, department, shift pattern, weekly overtime hours, night‐shift frequency, and the schedule notice period. Nurses who lived alone with children, had a child aged 16–18, with weekly overtime more than 8 h, and received schedules only 1–3 days in advance showed significantly higher turnover intention. Participants scored 12.50 ± 7.22 on the maternal feeling of childcare burden scale, 2.41 ± 0.51 on the practice environment scale, 55.74 ± 11.88 on the work–family conflict scale, and 15.20 ± 4.89 on the turnover intention scale. Higher work–family conflict was associated with greater turnover intention (*B* = 0.118, SE = 0.023, *β* = 0.282, *p* < 0.001) after controlling for the nursing practice environment.

**Conclusions:**

Among female nurses with children under 18 years of age, work–family conflict significantly mediated between the nursing practice environment and turnover intention. Reducing overtime and improving schedule predictability were critical, particularly for nurses living alone with their children and those with adolescents. Resources should foster supportive workplace culture that enhances task efficiency to manage workloads and minimize excessive overtime. Beyond the hospital, long‐term cultural change promoting equitable distribution of domestic labor is essential.

## 1. Introduction

The global nursing shortage presents a critical challenge to the sustainability of healthcare systems, with direct implications for patient safety, quality of care, and service delivery [1, 2]. Although estimates vary by region and study context, recent meta‐analytic evidence indicates that nursing staff turnover remains substantial worldwide, with pooled turnover rates estimated at approximately 20% in Asia, 15% in North America, and 7% in Europe [3]. In addition, 15 EU countries reported a shortage of nurses in 2022 and 2023 [4]. These findings suggest that nurse turnover and nurse shortages are shared workforce challenges across healthcare systems. In Japan, the turnover rate of hospital nurses has remained at approximately 10% over the past decade [5]. Globally, women constitute approximately 90% of the nursing workforce [6], and in aging societies like Japan, women of childbearing age form the essential core of this workforce [7]. In this group, the three leading reasons for leaving employment are marriage, childbirth, and parenting [8], which collectively result in a substantial loss of intellectual capital and productivity [9]. Nurse turnover not only depletes valuable clinical expertise and disrupts continuity of care but also exacerbates workloads and overtime burdens for the remaining staff, thereby fueling a costly cycle of recruitment and training [10]. Evidence has shown that for every one‐unit increase in workload, in‐hospital mortality rates can increase by up to 14% [11]. Conversely, a policy experiment in Queensland, Australia, demonstrated that reducing the patient‐to‐nurse ratio by one patient per nurse led to a 7% reduction in mortality and readmissions and a 3% reduction in the length of stay [12]. Thus, nurse turnover is not only a human‐resource issue but also a major factor affecting patient safety. These findings highlight the urgent need to address the determinants of nurses’ intent to leave, particularly among female nurses who are balancing professional roles with parenting responsibilities.

The nursing practice environment is defined as “the set of characteristics of the work context that facilitate or constrain professional nursing practice” [13] and is a well‐established predictor of nurses’ turnover intention (TI) [14]. It is often operationalized using five measurable dimensions: nurse participation in hospital affairs, nursing foundations for quality of care, nurse manager ability, leadership, and support of nurses, staffing and resource adequacy, and collegial nurse–physician relations. These characteristics directly shape the daily work experience of nurses by determining the extent to which they receive organizational support and professional autonomy in clinical practice. A study examining nursing practice environments in relation to retention‐related outcomes in Australia, including intent to leave, indicated that the practice environment is an important organizational factor influencing workforce stability [15]. Evidence from the United States further suggested that nurses’ TI is associated with work‐related resource depletion [16]. Recent European longitudinal evidence has examined the nursing practice environment in relation to nurse retention, showing that the pathway from organizational work environments to TI may be complex and requires further investigation [17]. In Japan, nurses frequently work in hospital environments marked by chronic workforce shortages, which lead to excessive workloads, frequent overtime, and stress levels reported to be higher than those of nurses in other countries [18]. These conditions may reflect insufficient organizational support within the practice environment, particularly in terms of staffing and resource adequacy. When such support is limited, work demands may systematically deplete nurses’ time, energy, and psychological resources.

Within the family domain, nurses with young children face substantial demands on their personal resources. Data have shown that Japanese women with children under 6 years of age spent an average of 7 h and 28 min per day on household and childcare duties, far exceeding the 1 h and 54 min contributed by their male partners [19]. This persistent unequal division of household chores and childcare responsibilities creates substantial demands in the family domain and further depletes personal resources. The convergence of intense work and family demands provides a fertile ground for work–family conflict (WFC). Greenhaus and Beutell [20] defined WFC as “a form of inter‐role conflict in which the role pressures from the work and family domains are mutually incompatible.” The work–home resources (W‐HR) model [21] explains how this conflict develops by describing how sustained workplace demands deplete personal resources and impede achievements in the family domain. Consequently, nurses who work shifts while raising children, whose parenting responsibilities require intensive time and emotional investment, are highly susceptible to an increased level of WFC [22].

Personal resources, together with family support (for example, spousal support, shared household chores, and caregiving responsibilities) and workplace support (for example, support from supervisors and colleagues and supportive nursing practice environment), function as protective factors against WFC among women [23, 24]. When these forms of support are insufficient and demands from both domains deplete personal resources, turnover may be perceived as the only viable strategy to prevent further depletion. Therefore, when the process of resource depletion exceeds the capacity of an individual to cope, TI can represent a direct behavioral manifestation. Current evidence indicates that a higher level of WFC is consistently associated with stronger TI among nurses [25]. Importantly, WFC itself acts as a mediator that translates the effects of inadequate family and workplace support into TI. Although the association between WFC and TI has been well established, there is limited international evidence on how specific family and workplace factors influence TI through the mechanism of WFC, particularly among nurses who are parenting children under 18 years of age. Identifying these factors and pathways is critical for developing targeted strategies to reduce turnover in the nursing workforce.

Therefore, this nationwide study aims to investigate the associations between key family and work environment factors and TI among female nurses parenting children under 18 years of age in general hospitals in Japan, with a specific focus on testing WFC as a mediator. We proposed the following research objectives: (a) to describe the level of TI and its associated personal, family‐related, and work‐related factors among female nurses in Japan with at least one child aged 18 years or younger; (b) to examine the direct relationships between the nursing practice environment, WFC, and TI; and (c) to establish the mediating role of WFC in the pathway from the nursing practice environment to TI. By examining this pathway in a nationwide Japanese sample, this study contributes to the global health workforce literature by clarifying a modifiable organizational pathway through which the nursing practice environment may influence TI via WFC among female nurses with parenting responsibilities.

## 2. Methods

### 2.1. Participant Recruitment and Sample Size

The participants eligible for this study included registered female nurses who had at least one child aged 18 years or younger. Participants who had been on leave for more than 1 month (excluding maternity leave) were excluded. Participants working 20 h or less per week were also excluded. Sample size estimation followed Kendall’s criterion for cross‐sectional studies (5–10 participants per survey domain). Given 29 core domains in the questionnaire, a sample size of 145–290 participants was required. Allowing for approximately 10% invalid responses, a target of 160–319 completed questionnaires was set.

### 2.2. Data Collection

A nationwide online survey was conducted in collaboration with NEO Marketing Inc., a certified market research company established in the year 2000. The company implemented a secure online questionnaire based on an instrument of the research group. Participants were randomly recruited from the company’s panel and invited to complete the survey. All information regarding the study and participants’ rights was provided on the first page of the survey. Consent items equivalent to those on paper‐based consent forms were presented, and participants were required to endorse all items before proceeding to the next page. Regarding the right to withdraw data, participants were free to discontinue or exit the survey at any point during its completion, in which case their partial responses were not recorded. However, once the questionnaire was submitted, it was no longer possible to withdraw the data because data collection was conducted entirely anonymously, with no mechanism to identify or link the submitted information to the personal identity of any individual. As an incentive for participation, respondents were eligible to receive premium points for shopping and other uses. Following data collection, the deidentified dataset was transmitted to the principal investigator (L.Y.Z.) via encrypted email. Data security protocols included password‐protected storage on a dedicated computer within Hiroshima University, Japan.

### 2.3. Measures

#### 2.3.1. Demographic Characteristics

Among female nurses with at least one child aged 18 years or younger, the following demographic and work‐related characteristics were assessed: age, cohabitation status, number of children, youngest child’s age, department, years of working experience, shift pattern, position, commuting time, weekly working hours, weekly overtime hours, night‐shift frequency, and schedule notice period. In addition, spouse‐related variables were recorded, including spouse’s occupational category, proportional division of household chores, spouse’s weekly childcare hours, number of shared days off per month with the spouse, and daily conversation time with the spouse.

#### 2.3.2. Maternal Feeling of Childcare Burden Scale

The feeling of childcare burden of the respondents was measured using an 8‐item Japanese‐language scale for maternal child‐rearing burden developed by Nakajima et al. [26]. The original validation study was conducted among public daycare centers and demonstrated good internal consistency with Cronbach’s alpha values of 0.819 for the total scale. Exploratory factor analysis identified a two‐factor structure, consisting of perceived restriction of activities and negative feelings, with good model fit in confirmatory factor analysis. It has subsequently been applied in Japanese mothers of preschool‐aged children [27]. Items were rated on a 0–4‐point scale: *never* (0), *occasionally* (1), *sometimes* (2), *often* (3), and *always* (4). The total score ranges from 0 to 32, with higher scores indicating a greater sense of child‐rearing burden. In this study, the scale demonstrated good internal consistency, with a Cronbach’s alpha value of 0.912.

#### 2.3.3. The Practice Environment Scale of the Nursing Work Index (PES‐NWI)

The nursing practice environment was measured using the Japanese version of the PES‐NWI [28]. The PES‐NWI‐J, translated from the original English version [13] by Ogata et al., comprises 31 items scored on a 4‐point Likert scale (1 = *strongly disagree* to 4 = *strongly agree*) to assess five components of the work environment: nurse participation in hospital affairs (nine items), nursing foundations for quality of care (10 items), nurse manager ability, leadership, and support of nurses (five items), adequacy of staffing (four items), and collegial nurse–physician relations (three items). The Japanese validation study supported its content validity and criterion‐related validity, provided partial evidence of factorial validity, and reported good internal consistency, with Cronbach’s alpha coefficients of 0.90 for the composite scale and 0.76–0.86 for the subscales. It has also been applied in a subsequent large study of Japanese university hospital nurses, in which the assessed subscales showed satisfactory internal consistency [29]. Mean value rather than total scores is used, which allows comparisons of score magnitudes across subscales. The overall score is calculated as the mean of the five subscale scores and ranges from 1 to 4, with a median of 2.5; higher scores indicate a more favorable professional nursing practice environment. In this study, the scale showed high reliability, with a Cronbach’s alpha of 0.948.

#### 2.3.4. WFC Scale

WFC was measured using the Japanese version of the WFC scale. The Japanese version, translated from the original English scale [30] by Watai et al. [31], was supported by confirmatory factor analysis, which confirmed the six‐factor structure of the scale. The validation study also reported discriminant validity, theoretically expected associations with external variables, and satisfactory reliability. Cronbach’s alpha coefficients for the subscales in previous research ranged from 0.77 to 0.92, indicating satisfactory internal consistency. The reliability of the scale was further supported in a subsequent study of Japanese female nurses with children [32]. Responses are rated on a 5‐point scale (1 = *strongly disagree*, 5 = *strongly agree*). The overall score ranges from 1 to 5 and is calculated as the mean of all items with higher scores indicating greater WFC. In this study, the Cronbach’s alpha values for this scale were 0.913.

#### 2.3.5. TI Scale

TI is defined as “the tendency to change jobs or leave one’s current job.” The TI of respondents was assessed as an outcome variable using a 6‐item scale developed by Tei et al. [33]. The scale was developed from Japanese workers, supporting the unidimensional structure of the 6‐item scale, with one factor explaining 68.48% of the variance and factor loadings ranging from 0.80 to 0.84. The original scale demonstrated high internal consistency, with a Cronbach’s alpha coefficient of 0.91. Items were rated on a 1–4 point scale: *never* (1), *seldom* (2), *sometimes* (3), and *frequently* (4). The total score ranges from 6 to 24, with higher scores indicating a stronger intention to leave. In this study, the scale demonstrated high reliability, with a Cronbach’s alpha of 0.914. Permission to use all scales was obtained from the original authors.

### 2.4. Statistical Analysis

All analyses were conducted using SPSS (Version 26.0; IBM Corp.) and the Process 4.0 macro plug‐in. Continuous variables are presented as mean ± standard deviation (M ± SD), and categorical variables are presented as frequencies and proportions. Group differences in TI across categorical sociodemographic factors were examined using one‐way analysis of variance or *t*‐tests, as appropriate. Associations among the maternal feeling of childcare burden, the nursing practice environment, WFC, and TI were assessed using Pearson correlation analysis. Subsequently, all variables that demonstrated significance (*p* < 0.05) in these univariate analyses were entered as independent variables into a multivariable linear regression model to identify key factors associated with TI. Further, to test the hypothesized mediating role of WFC in the relationship between the nursing practice environment and TI, a simple mediation analysis was conducted using Process Macro Model 4 with 5000 bias‐corrected bootstrap samples to generate 95% confidence intervals (CIs) values. For all analyses, statistical significance was set at a two‐tailed *p* value of less than 0.05.

### 2.5. Ethical Approval

This study was approved by the Hiroshima University Ethics Committee (Ethical Approval Number: E2025‐0043). All research activities were conducted in accordance with local Japanese legislation and institutional requirements. Informed consent was obtained from all participants prior to their participation in the study.

## 3. Results

### 3.1. Descriptive Statistics and Univariate Analysis

A total of 305 valid responses were obtained between July and September 2025 from nurses in 45 prefectures across Japan. The sociodemographic characteristics of the participants are summarized in Table 1. The mean age of nurses was 40.37 ± 7.27 years. With respect to childcare responsibilities, 110 nurses had one child, 136 nurses had two children, and 59 nurses had three or more children. Use of institutional support policies varied: parental leave was most frequently utilized (*n* = 168), followed by short‐time work (≤ 6 h/day; *n* = 147), leave for children’s illness (*n* = 128), night‐shift reduction (*n* = 116), and flexible working hours (*n* = 44). A total of 82 nurses reported not using any of these support options. Participants scored 12.50 ± 7.22 on the maternal feeling of childcare burden scale, 2.41 ± 0.51 on the PES‐NWI, 55.74 ± 11.88 on the WFC scale, and 15.20 ± 4.89 on the TI scale.

**TABLE 1:** Descriptive statistics and univariate analysis of variables associated with turnover intention among female nurses with children under 18 years (*n* = 305).

Demographic factors	(Mean ± SD) or *N* (%)	TI(M ± SD)	*F*/*t*	*p* value
Age	40.37 ± 7.270	15.20 ± 4.889	0.334	0.564
≤ 30	23 (7.5)	15.26 ± 4.266		
31–40	135 (44.3)	14.93 ± 4.722		
> 40	147 (48.2)	15.40 ± 5.089		
Cohabitation status	305	15.20 ± 4.889	4.209	0.041^∗^
Living alone with child(ren)	40 (13.1)	17.10 ± 4.706		
Living with spouse and child(ren)	232 (76.1)	14.92 ± 4.847		
Living with parents, spouse, and child(ren)	23 (7.5)	14.39 ± 5.061		
Living with parents and children (without spouse)	10 (3.3)	15.40 ± 4.142		
Number of children	305	15.20 ± 4.889	0.182	0.670
1 child	110 (36.1)	15.13 ± 4.303		
2 children	136 (44.6)	15.08 ± 5.118		
3 children or more	59 (19.3)	15.53 ± 5.289		
The youngest child’s age	8.20 ± 5.614	15.20 ± 4.889	5.925	0.016^∗^
≤ 6	134 (43.9)	14.68 ± 4.520		
7–15	138 (45.2)	15.18 ± 5.221		
16–18	33 (10.8)	17.24 ± 4.184		
Working years	11.77 ± 7.082	15.20 ± 4.889	0.878	0.349
0–3 years	34 (11.1)	15.21 ± 5.969		
4–10 years	117 (38.4)	14.77 ± 4.578		
> 10 years	154 (50.5)	15.49 ± 4.810		
Department	305	15.20 ± 4.889	4.197	0.041^∗^
Outpatient and others	117 (38.4)	14.81 ± 4.518		
Internal medicine/surgery	145 (47.5)	15.07 ± 5.145		
Emergency/ICU/obstetrics/pediatrics	43 (14.1)	16.58 ± 4.641		
Shift pattern	305	15.20 ± 4.889	7.065	0.008^∗^
Day shift	124 (40.7)	14.48 ± 4.621		
2‐shift	132 (43.3)	15.27 ± 5.016		
3‐shift	42 (13.8)	16.79 ± 4.946		
Emergency response	7 (2.3)	16.43 ± 3.645		
Position	305	15.20 ± 4.889	0.267	0.790
Head nurse	43 (14.1)	15.00 ± 4.865		
Clinical nurse	262 (85.9)	15.21 ± 4.870		
Commuting time	305	15.20 ± 4.889	1.042	0.308
< 30 min	202 (66.2)	15.33 ± 4.798		
30 min‐1 h	90 (29.5)	14.74 ± 5.194		
> 1 h	13 (4.3)	15.92 ± 3.278		
Weekly working hours	40.03 ± 7.393	15.20 ± 4.889	2.104	0.148
≤ 30 h	36 (11.8)	15.50 ± 4.359		
31–40 h	190 (62.3)	14.83 ± 4.815		
> 40 h	79 (25.9)	15.90 ± 5.153		
Weekly overtime hours	2.73 ± 3.232	15.20 ± 4.889	8.656	0.004^∗^
0 h	77 (25.2)	13.36 ± 4.853		
1–4 h	148 (48.5)	15.93 ± 4.583		
5 h–8 h	64 (21.0)	15.17 ± 5.048		
> 9 h	16 (5.2)	17.06 ± 4.538		
Night shifts frequency within a month	305	15.20 ± 4.889	5.316	0.022^∗^
No night shift	133 (43.6)	14.39 ± 4.582		
1–4 night shifts	99 (32.5)	15.71 ± 4.864		
5–8 night shifts	55 (18.0)	15.82 ± 5.302		
> 9 night shifts	18 (5.9)	16.22 ± 5.001		
Schedule notice period	305	15.20 ± 4.889	4.952	0.027^∗^
Published 1–3 days ago	18 (5.9)	17.78 ± 4.697		
Published within 1 week	76 (24.9)	15.72 ± 5.127		
Published 1 week ago	170 (55.7)	14.71 ± 4.666		
Indeterminacy	41 (13.4)	15.02 ± 4.942		
Utilization of parenting support	305	15.20 ± 4.889	0.607	0.437
Not accepted	82 (26.9)	15.17 ± 4.883		
Accept 1 item	57 (18.7)	14.88 ± 4.822		
Accept 2 items	48 (15.7)	16.21 ± 5.036		
Accept 3 or more items	118 (38.7)	14.92 ± 4.801		

Abbreviation: TI, turnover intention.

^∗^ *p* < 0.05.

Univariate analyses revealed significant differences in TI according to cohabitation status, youngest child’s age, department, shift pattern, weekly overtime hours, night‐shift frequency, and schedule notice period. Notably, nurses who lived alone with their children, had a youngest child aged 16–18, worked more than 8 h of overtime per week, and received their work schedules only 1–3 days in advance reported significantly higher TI. In addition, TI differed significantly by night‐shift frequency, shift pattern, and department (details in Tables 1 and 2).

**TABLE 2:** Descriptive statistics and univariate analysis of variables associated with turnover intention among female nurses with spouses (*n* = 255).

Demographic factors	*N* (%)	Turnover intention (M ± SD)	*F*/t	*p* value
Spouse’s occupation type	255	14.87 ± 4.859	0.281	0.596
Fixed time jobs	128 (50.2)	14.90 ± 4.818		
Long‐hour jobs	96 (37.6)	15.00 ± 4.916		
Flexible‐hour jobs	31 (12.2)	14.39 ± 4.978		
Proportional division of household chores	255	14.87 ± 4.859	0.112	0.738
Mainly by myself	184 (72.2)	14.97 ± 5.058		
Self 5: Spouse 5	53 (20.8)	14.53 ± 4.449		
Mainly by spouse	18 (7.0)	14.94 ± 4.036		
Weekly childcare hours by spouse (average)	255	14.87 ± 4.859	0.916	0.339
< 1 h	67 (26.3)	14.91 ± 4.535		
1–5 h	114 (44.7)	14.54 ± 5.197		
> 5 h	74 (29.0)	15.35 ± 4.618		
Monthly shared days off with spouse	255	14.87 ± 4.859	1.304	0.255
0–2 days	112 (43.9)	15.04 ± 5.095		
3–4 days	90 (35.3)	14.42 ± 4.700		
> 5 days	53 (20.8)	15.30 ± 4.635		
Daily conversation time with spouse (average)	255	14.87 ± 4.859	0.601	0.439
< 30 min	90 (35.3)	14.91 ± 5.356		
30‐1 h	114 (44.7)	15.10 ± 4.434		
> 1 h	51 (20.0)	14.31 ± 4.897		

### 3.2. Pearson Correlation Analysis

WFC was positively correlated with the maternal feeling of childcare burden (*r* = 0.40, *p* < 0.01) and TI (*r* = 0.36, *p* < 0.01). In contrast, the nursing practice environment was significantly negatively correlated with WFC (*r* = −0.33, *p* < 0.01) and TI (*r* = −0.34, *p* < 0.01).

### 3.3. Multivariate Linear Regression Analysis

Table 3 presents the results of the multivariate linear regression analysis for TI. The overall model was significant (*F* = 5.638, *p* < 0.001) and explained 21.5% of the variance in TI (R^2^adj = 0.215). TI was significantly associated with weekly overtime hours, schedule notice period, the nursing practice environment, and WFC.

**TABLE 3:** Multiple linear regression analysis of variables associated with turnover intention among female nurses with children under 18 years (*n* = 305).

	*B*	Beta	*t*	*p* value	Tolerance	VIF	*F*	Adjust *R* ^2^
The youngest child’s age ≤ 6	0						5.638^∗∗^	0.215
The youngest child’s age 7–15	0.005	0	0.009	0.993	0.775	1.29		
The youngest child’s age 16–18	1.71	0.109	1.937	0.054	0.808	1.238		
Weekly overtime hours 0	0							
Weekly overtime hours 1–4 h	1.64	0.169	2.513	0.013^∗^	0.571	1.75		
Weekly overtime hours 5–8 h	0.77	0.065	0.952	0.342	0.561	1.783		
Weekly overtime hours > 9 h	2.548	0.117	2.044	0.042^∗^	0.787	1.27		
No night shift	0							
1–4 night shifts	0.932	0.09	0.858	0.392	0.235	4.261		
5–8 night shifts	0.426	0.034	0.34	0.734	0.263	3.804		
> 9 night shifts	−0.411	−0.02	−0.266	0.79	0.458	2.182		
Outpatient and others	0							
Internal medicine/surgery	−0.463	−0.048	−0.759	0.448	0.657	1.523		
Emergency/ICU/obstetrics/pediatrics	1.46	0.105	1.671	0.096	0.658	1.52		
Published 1–3 days ago	0							
Published within 1 week	−1.961	−0.175	−1.691	0.092	0.242	4.139		
Published 1 week ago	−2.522	−0.258	−2.279	0.023^∗^	0.201	4.97		
Indeterminacy	−3.055	−0.215	−2.453	0.015^∗^	0.337	2.969		
Day shift	0							
2‐shift	−0.611	−0.062	−0.559	0.577	0.207	4.823		
3‐shift	0.159	0.011	0.121	0.904	0.296	3.375		
Emergency response	1.017	0.031	0.571	0.568	0.854	1.171		
Nursing practice environment	−2.353	−0.245	−4.224	< 0.001^∗∗^	0.765	1.308		
Work–family conflict	0.096	0.228	3.951	< 0.001^∗∗^	0.772	1.295		

^∗^ *p* < 0.05, ^∗∗^ *p* < 0.001.

### 3.4. Mediation Analysis

A mediation analysis was conducted to examine the role of WFC in the association between the nursing practice environment and TI. First, regression analysis indicated that the nursing practice environment significantly and negatively predicted WFC (unstandardized beta [B] = −7.472, standard error [SE] = 1.245, *β* = −0.326, *t* = −6.000, *p* < 0.001), accounting for 10.6% of its variance (*F* = 36.004, *p* < 0.001). Second, the result of a multiple regression analysis predicting TI from both the nursing practice environment and WFC was significant (*F* = 34.982, *p* < 0.001), explaining 18.8% of the variance in TI. In this model, WFC remained a significant positive predictor of TI (*B* = 0.118, SE = 0.023, *β* = 0.282, *p* < 0.001) after controlling for the nursing practice environment. The direct effect of the nursing practice environment on TI remained significant but reduced in magnitude (*B* = −2.403, SE = 0.526, *β* = −0.251, *p* < 0.001). These findings indicate a significant indirect effect and support a partial mediating role of WFC in the relationship between the nursing practice environment and TI (details in Table 4).

**TABLE 4:** Mediation analysis of associations between the nursing practice environment, work–family conflict, and turnover intention among female nurses with children under 18 years (*n* = 305).

Outcome: Work–family conflict								
Variables	*R* ^2^	*F*	*B*	SE	*β*	*t*	LLCI	ULCI
Nursing practice environment	0.106	36.004	−7.472	1.245	−0.326	−6.000^∗∗^	−9.923	−5.022

**Outcome: turnover intention**								
**Variables**	***R*** ^***2***^	***F***	***B***	**SE**	**β**	***t***	**LLCI**	**ULCI**

	0.188	34.982						
Nursing practice environment			−2.403	0.526	−0.251	−4.568^∗∗^	−3.438	−1.367
Work–family conflict			0.118	0.023	0.282	5.136^∗∗^	0.073	0.163

^∗∗^ *p* < 0.001.

## 4. Discussion

This study examined the relationships between parental burden, the nursing practice environments, WFC, and TI among female nurses living with children under 18 years of age in Japan and tested the mediating role of WFC. The key findings confirmed significant associations among these variables, with WFC partially mediating the relationship between the nursing practice environment and TI.

The mean score for the nursing practice environment among female nurses with child‐rearing responsibilities in this study was 2.41 ± 0.51, which is slightly lower than that reported for the broader population of female nurses in Japan (2.60 ± 0.40) [34]. This disparity suggests that child‐rearing responsibilities are a potential key risk factor that exacerbates negative perceptions of the nursing practice environment. A plausible explanation is that standard nursing practice environments may not adequately accommodate the specific needs of nurses with childcare responsibilities, such as schedule flexibility. When organizational structures do not provide such flexibility, nurses are more susceptible to WFC, which in turn can lead to a more negative appraisal of their practice environment. The nursing practice environment score in this study was comparable to that reported among midwives in Spain (2.47 ± 0.43) [35]. Given that midwifery is characterized by high emotional labor, unpredictable work hours, and frequent exposure to traumatic events, the similarity between our findings and those from Spanish midwives suggests that female nurses with child‐rearing responsibilities face levels of practice environment that are on par with other high‐stress, female‐dominated healthcare professions. This parallel reinforces the interpretation that routine organizational support may be insufficient to offset the cumulative burden of childcare and demanding clinical work. Furthermore, a study in Italy reported that female nurses had slightly higher practice environment scores than their male counterparts (2.42 vs. 2.29) [36]. In our study, all participants were female nurses with children under 18, and their scores (2.41) were identical to the Italian female nurses’ average but substantially lower than the Japanese general female nurse average (2.60). This pattern indicates that being female alone does not explain the lower scores; rather, the added responsibility of active childcare is the key differentiator. Therefore, interventions aimed at improving the practice environment for nurses should go beyond gender‐neutral policies and specifically address the structural needs of nurses who are primary caregivers, such as predictable scheduling, on‐site childcare, and emergency backup support.

Participants in the present study showed a moderate level of WFC (55.74 ± 11.88), with a mean item score of 3.10 ± 0.66, which was somewhat higher than that reported in previous Japanese study involving nurses who are mothers (2.90 ± 0.50) [10], suggesting a potential increase in WFC in this population. Asian evidence [37, 38] has highlighted the substantial impact of parental roles on WFC, particularly among those with multiple children, financial strain, or health limitations. Recent European evidence points to a similar pattern. In an Italian multicenter study of nurses, work–family and family–work conflicts were associated with age and the presence of children and/or older family members requiring care, while work organization factors such as rostering and work articulation appeared to help prevent work–life conflict [22]. These findings, when considered alongside the moderate nursing practice environment scores, suggest that the roots of this conflict extend beyond the workplace. Even a moderately favorable nursing practice environment may still be insufficient to offset the distinct WFC experienced by female nurses with heavy childcare responsibilities. The elevated level of WFC observed in our sample is likely situated at the intersection of high familial demands and limited societal support structures. For instance, the well‐developed Japanese parental leave system has considerable transformative potential, but this potential has been diluted by low uptake among fathers [39], which has hindered a substantive realignment of domestic roles. This pattern suggests that policy interventions alone are insufficient to transform deeply rooted family norms. Furthermore, male‐centric workplace structures often limit the practical involvement of fathers in childcare, and the “mental load” for housework and childcare remains predominantly with mothers. Consequently, the inequitable distribution of domestic labor continues to sustain the high levels of WFC observed among nurse mothers.

Ultimately, the cumulative effect of these unresolved conflicts contributes to a high level of TI. The mean TI score for nurse mothers in this study (15.20 ± 4.89) was higher than mean values reported for general Japanese healthcare workers (11.00 ± 4.70) [40] and for nurses in long‐term care facilities (13.76 ± 5.21**)** [41]. Notably, it was comparable to levels observed in high‐intensity specialties such as pediatrics (15.5 ± 5.3) [42]. This finding indicates that for nurse mothers, the combination of a demanding profession and intensive childcare responsibilities creates a dual‐pressure effect that places them at similarly high risk of leaving the profession as nurses working in the most stressful specialties. We interpret this phenomenon as a direct consequence of the WFC described above, reflecting the tension between entrenched sex‐related role expectations and a slowly evolving social context. Paternity leave uptake in Japan remains low, in part because of unsupportive work environments [43]. Recent data, including increased male participation in domestic tasks [19] and a reported paternal leave uptake rate of 40.5% in 2024 [44], indicate that current developments represent incremental “quantitative changes” that have not yet led to a “qualitative” redistribution of responsibility. The absolute increase in male domestic hours often remains marginal, and the duration of paternal leave is frequently short. Consequently, the long‐term burden of childcare and family responsibilities continues to fall predominantly on women. Nurse mothers are caught in a disconnect in which societal expectations for gender equality are rising, yet practical support from both family and workplace lags behind. This gap between progressive ideals and stagnant realities exacerbates their role tension and frustration, ultimately fostering the strong TI level observed. In summary, the high TI among Japanese nurse mothers signals that policy implementation, although encouraging, must be accompanied by sustained efforts to address deep‐rooted cultural norms that shape both domestic and workplace roles.

### 4.1. The Interplay of Nursing Practice Environment, WFC, and TI

Our current findings confirmed that both an unfavorable nursing practice environment and high WFC are directly associated with increased TI, consistent with previous international evidence [45, 46]. Recent evidence from a five‐country study of critical care nurses showed that 22.8% of nurses planned to leave their position, and intention to leave was associated with poorer perceptions of the work environment [47]. A central finding is the role of WFC as a significant mediator between the nursing practice environment and TI. This extends prior research [29] that underscored the relevance of the practice environment for the personal career development of nurses. Our mediation model delineates an underlying psychological pathway: An unfavorable practice environment directly hinders career progression and is also likely to deplete the personal resources of nurses, ultimately fostering an intention to leave. The job demands–resources model provides a plausible theoretical framework for interpreting this phenomenon [48]. When job demands are high and organizational resources are limited, as occurs in unfavorable practice environments, nurses are compelled to mobilize both work‐ and family‐related resources to maintain work–family balance. Persistent work overload combined with substantial domestic responsibilities can deplete personal resources, thereby precipitating WFC [49] and, ultimately, a stronger intention to leave the profession. The implications of an elevated TI extend beyond individual career trajectories and represent a considerable organizational financial burden and, as underscored by Brborović et al. [50], a serious threat to patient safety and quality of care. For healthcare managers, this implies that optimizing the nursing practice environment is essential for mitigating TI. Key strategies include strengthening organizational support structures, fostering an inclusive and empowering workplace culture, and implementing more flexible and predictable scheduling systems. Such systemic improvements would protect the well‐being of nurses and support the long‐term stability and sustainable development of the nursing workforce. In parallel, efforts should address the roots of role conflict by reinforcing family support systems and challenging the gendered distribution of domestic labor, which are crucial for reducing WFC.

### 4.2. Limitations

This study has several limitations that should be considered. First, the cross‐sectional study design limits causal inference. Although the proposed model is theoretically grounded, longitudinal or diary‐based studies are needed to verify the causal pathways and clarify the temporal dynamics through which perceptions of the practice environment influence WFC and TI over time. Second, all measures were self‐reported and therefore susceptible to reporting and recall biases. Future studies could incorporate objective data (for example, actual turnover rates, economic indicators, or the availability of grandparental support) to enhance the robustness of the findings. Third, despite the nationwide recruitment of participants, the sample size (*n* = 305) may have limited the statistical power to detect more complex relationships and may affect the generalizability of the results. Expanding the sample size and employing multicenter sampling in future work would strengthen the evidence. Finally, assessments of the nursing practice environment may have been influenced by social desirability bias, as nurses might have provided responses they perceived to be more favorable.

### 4.3. Policy Implications

Our findings, which identify WFC as a critical mediator between the nursing practice environment and TI, yield specific and actionable implications. A multilevel strategy is required to address the turnover of nurses. At the institutional level, healthcare facilities can draw on the framework provided by the 2023 directive of the Japanese Ministry of Health, Labour and Welfare [51] on improving the nursing practice environment. Our results support three priority areas for action. First, institutions should implement a comprehensive support system that integrates childcare support with flexible work arrangements. When institutional resources in terms of funding and space permit, on‐site childcare services could directly alleviate family‐domain pressures and reduce time‐based strain associated with commuting. In addition, broader adoption of flexible work schedules and strategic hiring of part‐time staff are recommended so that these staff can provide coverage when mothers need to take sudden leave. Such flexible staffing can help ensure operational continuity in hospital units and reduce the psychological burden on nurses when they must be absent for family reasons. Second, institutions should promote nursing task efficiency to reduce excessive workloads and overtime, thereby helping nurses conserve personal resources. Third, facilities should allocate funding to workplace culture initiatives that foster a positive nursing practice environment, including supportive leadership and the implementation of concrete policies such as enhanced schedule predictability and strict limits on overtime. Beyond the hospital, it is important to support long‐term cultural shifts that redistribute domestic labor. Although this represents a structural challenge, policymakers could initiate public campaigns that normalize shared parenting and household responsibilities in order to create a more supportive environment for nurses.

## 5. Conclusion

This study established a significant mediation model, indicating that improving the nursing practice environment and reducing WFC can effectively lower TI among female nurses during their child‐rearing years. Among Japanese nurses with children aged 18 years or younger, overtime hours and schedule inflexibility emerged as key proximal predictors of TI, whereas family‐related factors influenced TI indirectly through WFC. These findings highlight the critical need for healthcare administrators to implement targeted interventions. Enhancing schedule control, reforming overtime policies, and fostering a supportive and inclusive organizational culture are essential and cost‐effective strategies to mitigate turnover risk in this vital segment of the nursing workforce. Such measures are indispensable to safeguard the well‐being of nurses, maintain the stability of the healthcare system, and ultimately protect patient safety.

## Author Contributions

Liyuan Zheng: writing–original draft, writing–review and editing, visualization, validation, methodology, investigation, formal analysis, data curation, and funding acquisition. Hiromi Kawasaki: supervision, project administration, conceptualization, and writing–review and editing. Wenjie Guo: formal analysis, data curation, and writing–review and editing. Miyuki Toda: methodology and writing–review and editing. Yoko Shimpuku: supervision, survey resources, project administration, conceptualization, and writing–review and editing.

## Funding

This work was supported by JST SPRING (Grant Number JPMJSP2132) and the JSPS Program for Forming Japan’s Peak Research Universities (J‐PEAKS; Grant Number JPJS00420230011).

## Ethics Statement

This study was approved by the Hiroshima University Medical Ethics Committee (Ethical Approval Number: E2025‐0043). All research activities were conducted in compliance with local Japanese legislation and institutional requirements. Informed consent was obtained from all participants prior to their involvement in this study.

## Conflicts of Interest

The authors declare no conflicts of interest.

## Supporting Information

Additional supporting information can be found online in the Supporting Information section.

## Supporting information


**Supporting Information** STROBE Checklist is provided in the supporting section.

## Data Availability

The data that support the findings of this study are available on request from the first author.
